# False Negative Cell-Free DNA Screening Result in a Newborn with Trisomy 13

**DOI:** 10.1155/2016/7397405

**Published:** 2016-02-22

**Authors:** Yang Cao, Nicole L. Hoppman, Sarah E. Kerr, Christopher A. Sattler, Kristi S. Borowski, Myra J. Wick, W. Edward Highsmith, Umut Aypar

**Affiliations:** ^1^Department of Laboratory Medicine and Pathology, Mayo Clinic, Rochester, MN 55905, USA; ^2^Department of Obstetrics and Gynecology, Mayo Clinic, Rochester, MN 55905, USA

## Abstract

*Background.* Noninvasive prenatal screening (NIPS) is revolutionizing prenatal screening as a result of its increased sensitivity, specificity. NIPS analyzes cell-free fetal DNA (cffDNA) circulating in maternal plasma to detect fetal chromosome abnormalities. However, cffDNA originates from apoptotic placental trophoblast; therefore cffDNA is not always representative of the fetus. Although the published data for NIPS testing states that the current technique ensures high sensitivity and specificity for aneuploidy detection, false positives are possible due to isolated placental mosaicism, vanishing twin or cotwin demise, and maternal chromosome abnormalities or malignancy.* Results.* We report a case of false negative cell-free DNA (cfDNA) screening due to fetoplacental mosaicism. An infant male with negative cfDNA screening result was born with multiple congenital abnormalities. Postnatal chromosome and FISH studies on a blood specimen revealed trisomy 13 in 20/20 metaphases and 100% interphase nuclei, respectively. FISH analysis on tissues collected after delivery revealed extraembryonic mosaicism.* Conclusions.* Extraembryonic tissue mosaicism is likely responsible for the false negative cfDNA screening result. This case illustrates that a negative result does not rule out the possibility of a fetus affected with a trisomy, as cffDNA is derived from the placenta and therefore may not accurately represent the fetal genetic information.

## 1. Background

Cell-free fetal DNA (cffDNA) circulating in maternal plasma was initially identified approximately two decades ago; however, the laboratory use of cffDNA to detect fetal chromosome abnormalities was not available until 2011 [1]. cfDNA screening (also referred to as NIPS, NIPT) analyzes cffDNA circulating in maternal plasma. Clinical utilization of cfDNA screening has been rapidly incorporated into obstetric practice, as it offers improved sensitivity, specificity, and PPV when compared to first- and second-trimester screening. However, cfDNA screening also has limitations. The cffDNA originates from apoptotic placental trophoblast cells [2]; therefore, cffDNA may not always represent the chromosomal make-up of the fetus. Although the genetic component of placental and fetal tissue is identical in the vast majority of pregnancies, false positive or false negative results may be due to fetoplacental mosaicism. In several reported cases, follow-up amniocentesis based on positive cfDNA screening results has identified a normal karyotype, suggesting a false positive cfDNA screening result [3–8]. Even though the published data indicates high sensitivity (≥99% for trisomy 21, ≥92% for trisomy 18, and ≥87% for trisomy 13) and specificity (≥99% for trisomy 21, 18, and 13) for aneuploidy detection [9], false positive results have been reported for confined placental mosaicism, vanishing twin or cotwin demise, fetal chromosome rearrangement, and maternal chromosome abnormalities or malignancy [10–14]. Based on several reports, false negative results for fetal aneuploidy are much less common than false positive results [4–6, 15, 16]. It is generally accepted that false negative cfDNA screening results are primarily due to a low level of cffDNA fraction in maternal plasma and therefore could be overcome by technical improvement [17]. However, aside from the technical reasons, a limited number of false negative cfDNA screening cases due to fetoplacental mosaicism and/or structural chromosome rearrangement have also been reported [13, 18].

## 2. Case Presentation

A 19-year-old, gravida 2, para 1, female underwent obstetric ultrasound at 19 5/7 weeks of gestation, which identified multiple fetal anomalies including hypoplastic left heart, bilateral cleft lip, bilateral echogenic kidneys with hydronephrosis, echogenic bowel, and bowed right femur. Genetic consultation was provided and risks, benefits, and alternatives of further genetic evaluation, including amniocentesis and cfDNA screening, were discussed. The patient expressed concerns regarding the risks of invasive testing and opted to proceed with cfDNA screening. Limitations of cfDNA in this setting were reviewed. cfDNA screening was performed at 20 weeks of gestational age. A negative cfDNA screening result was issued for chromosomes 13, 18, 21, X, and Y. Although the fetal fraction (percentage of fetal DNA among all DNA in maternal plasma) was not included in the final report, later inquiries to the testing laboratory revealed a fetal fraction of 8.5%. Genetic counseling was provided to the patient at 24-5/7 weeks of gestation, during which amniocentesis with cytogenetic analyses was further discussed. The patient again declined invasive testing. After induction of labor due to multiple fetal anomalies, a male infant was delivered vaginally at 38-4/7 weeks of gestational age. Apgar scores were 8, 7, and 9 at one, five, and ten minutes, respectively. Physical examination revealed multiple anomalies including cutis aplasia on the scalp, cleft lip and palate, polydactyly, and cryptorchidism; postnatal echocardiogram confirmed hypoplastic left heart. The newborn also had respiratory insufficiency and was intubated due to signs of airway obstruction. A peripheral blood specimen was collected at birth and sent to the Cytogenetics Laboratory for postnatal evaluation.

**Figure 1:**
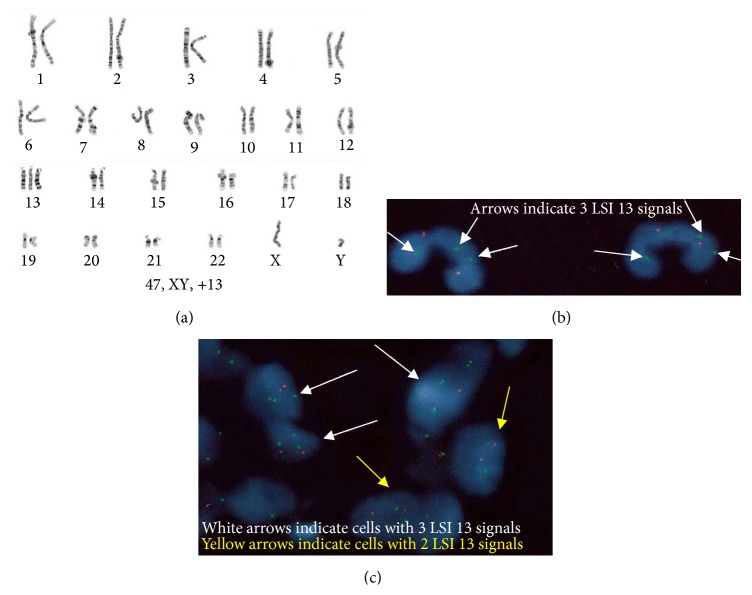
Postnatal aneuploidy detection by chromosome and FISH analysis. (a) G-banded karyotype on a blood specimen of the newborn shows trisomy 13. (b) FISH analysis on a blood specimen of the newborn shows three signals of LSI 13 (RB1) probe targeted on 13q14 (green) and two signals of LSI 21 (D21S341) probe targeted on 21q22.13-q22.2 (orange). Cells are stained with DAPI (blue) to visualize nuclei. (c) FISH analysis for aneuploidy detection in different tissue types of placental specimen shows mosaic trisomy 13. Signals of LSI 13 (RB1) probe targeted on 13q14 are shown in green. Signals of LSI 21 (D21S341) probe targeted on 21q22.13-q22.2 are shown in orange. Cells are stained with DAPI (blue) to visualize nuclei.

Chromosome analysis was performed on 20 metaphases, which identified additional chromosome 13 in each metaphase (47, XY, +13), suggesting nonmosaic trisomy 13 (Figure 1(a)). FISH analysis was also performed, and 100% of nuclei indicated three signals of probes for chromosome 13, also consistent with nonmosaic trisomy 13 (Figure 1(b)). Unfortunately, the infant passed away four days after birth.

We proposed that the discordant cfDNA and postnatal cytogenetic results were due to fetoplacental mosaicism. To test this hypothesis, we further evaluated extraembryonic tissue samples. The placenta was of normal weight (588 grams) and gross appearances of the placenta and umbilical cord were normal for 38 weeks of gestation. The chorionic villi showed mild villous enlargement and edema with increase in Hofbauer cells, but no other morphologic abnormalities. Representative areas of the umbilical cord, amnion, villous trophoblast, villous stroma, and intermediate trophoblast were identified from placental sections for aneuploidy analysis by FISH. As shown in Figure 1(c), all five types of extraembryonic tissues demonstrated different levels of extraembryonic mosaicism of trisomy 13 (58%, 57%, 32%, 46%, and 64% of nuclei with trisomy 13 in umbilical cord, amnion, villous trophoblast, villous stroma, and intermediate trophoblast, resp., data listed in Table 1). These results suggest that mosaicism of the extraembryonic tissues was responsible for the false negative cfDNA screening result in this case.

## 3. Materials and Methods

### 3.1. G-Banding

Peripheral blood lymphocytes were cultured for 72 h in PB-Max plus excess thymidine and were harvested according to standard cytogenetic protocols. Metaphases were dropped in a Thermotron chamber and baked for 1 hour and 30 minutes at 100°C. G-banding was performed according to standard cytogenetic methods using trypsin and Leishman stain. Twenty GTL-banded metaphases were evaluated.

### 3.2. Fluorescence* In Situ* Hybridization (FISH)

Initial FISH for newborn aneuploidy detection was performed on a peripheral blood sample. Additional postpartum FISH analysis for aneuploidy detection was performed on formalin fixed paraffin-embedded tissue. We used FISH probes that hybridize to the X centromere (DXZ1), Y centromere (DYZ3), 13q14 (Rb1), 13q34 (LAMP1), 18 centromere (D18Z1), and 21q22 (D21S341). Bacteria artificial chromosomes (BACs) located within the critical regions listed above were used for FISH probe development. Briefly, each BAC was extracted from* E. coli* using the Qiagen Plasmid Maxi kit according to the manufacturer's instructions and was then labeled with SpectrumOrange, SpectrumGreen, or SpectrumAqua (Abbott Molecular) using the Nick Translation Kit (Abbott Molecular) according to the manufacturer's instructions. A FISH probe working solution was made by adding 3 *μ*L of labeled BAC to 7 *μ*L of LSI/WCP® hybridization buffer (Abbott Molecular). Slides were pretreated according to standard cytogenetic protocols followed by application of 3 *μ*L of probe working solution to the hybridization site. Slides were denatured at 75°C for 5 min and hybridized at 37°C for 70 h followed by washing for 2 min at 72°C with 0.4x saline-sodium citrate (SSC) and rinsed in 0.1% NP-40/2xSSC for 1 min at room temperature. 10 *μ*L counterstain [10% 4′,6-diamidino-2-phenylindole (DAPI)] was added to the hybridization area. One hundred nuclei were scored for each specimen for aneuploidy detection.

## 4. Discussion

cfDNA screening is revolutionizing prenatal screening as a result of its robust test performance with increased sensitivity and specificity for the detection of common autosomal aneuploidies (trisomies 13, 18, and 21) compared to other prenatal aneuploidy screening methodologies. Since the introduction in the clinical setting, there have been several reports of cfDNA performance in high-risk population (indicated by advanced maternal age, screen positive on first- or second-trimester serum biochemical screening, the presence of a fetal abnormality on ultrasound, or a personal or family history of a chromosomal abnormality) as well as in low-risk population (general population) [4, 5, 9]. In high-risk pregnancies, cfDNA screening PPV for common autosomal aneuploidies varies from 90.9% to 100% based on different studies, while NPV remains as 99.9%–100% [4, 5, 9]. In low-risk pregnancies, PPV for common autosomal aneuploidies has been reported as 85.3% with 99.9% NPV based on 146,958 pregnancies [4]. Thus, although clinical performance of cfDNA screening is widely accepted and desirable for screening utility, it is not appropriate for use as a diagnostic test.

Some of the reasons for false positive and negative cfDNA screening results include fetoplacental mosaicism, a vanishing twin/cotwin demise, maternal chromosome abnormality, and maternal metastatic disease. Several published reports of false positive and false negative cfDNA screening cases have implicated fetoplacental mosaicism [3, 8, 13, 18]. The most common type of fetal-placental mosaicism, confined placental mosaicism (CPM), is a conception with normal fetus and placenta mosaic for a chromosome abnormality [19]. However, false negative cfDNA screening results cannot be explained by CPM. Instead, it may be due to another type of fetoplacental mosaicism in which there is an affected (possibly nonmosaic) fetus and mosaic or normal placenta. Although false negative cfDNA screening result for trisomy 13 has been reported at least once, the causative mechanism of false negative result was not identified at that time [20]. This is the first report of a case with false negative cfDNA screening result for trisomy 13 that is caused by fetoplacental mosaicism. For trisomies 18 and 21, this mechanism has been recently described. A false negative trisomy 18 cfDNA screening result due to 48, XXX, +18 placental mosaicism and two cases with false negative trisomy 21 due to placental mosaicism have been reported [3, 13]. Potential contribution of fetoplacental mosaicism to discordant cfDNA screening results has been reported; this study suggested that the sensitivity or specificity of cfDNA screening will never reach 100% due to the nature of fetoplacental mosaicism.

This report describes a case of false negative cfDNA results for trisomy 13 due to fetoplacental mosaicism. Cytogenetics analysis demonstrated that the infant was nonmosaic for trisomy 13 with placental tissue mosaic for trisomy 13. This mosaicism was demonstrated in at least five different extraembryonic tissues including umbilical cord, amnion, villous trophoblast, villous stroma, and intermediate trophoblast. In this case, the level of trisomy 13 mosaicism in the various tissues investigated ranged from 32% to 64%. The level of fetal fraction required for robust detection of trisomies varies slightly among commercial providers but is generally in the range of 4%. In the case presented here, the proportion of the 8.5% fetal fraction that derived from trisomy 13 positive extraembryonic tissues was likely less than half, thus decreasing the functional fetal fraction to less than the level required. Similar patterns of mosaicism have been reported in cases with false negative cfDNA screening results [3, 13, 18]. This type of fetoplacental mosaicism is different from CMP, which is more commonly reported etiology for discordant cfDNA and fetal/infant cytogenetic results. Cytogenetic analyses of infant cord blood as well as physical examination suggested nonmosaic trisomy 13 for the infant. However, because our studies were limited to a single specimen type from the infant, we cannot rule out the possibility that the infant may have had mosaicism in other tissues. This case, along with other reports of discordant cfDNA screening findings, demonstrates that providers should understand and patients should be counseled regarding the limitations of cfDNA screening. In the setting of a positive cfDNA screening result, confirmatory diagnostic testing is highly recommended. This case report also underscores the importance of diagnostic testing in the setting of multiple fetal anomalies with negative cfDNA results. Pre- and posttest genetic counseling are important in such cases to educate about the benefits and limitations of cfDNA screening to ensure that patients are able to make informed decisions.

## 5. Conclusions

Here we report a case with negative cfDNA screening results that had a postnatal diagnostic test result of nonmosaic trisomy 13. FISH analysis on placental tissues revealed extraembryonic mosaicism of trisomy 13, which is likely responsible for the false negative cfDNA screening result. This case illustrates the limitation of cfDNA screening, as cell-free fetal DNA is derived from the placenta and therefore may not accurately represent the fetal genetic information. Therefore, in the setting of multiple fetal anomalies with negative cfDNA screening results, diagnostic testing is recommended.

## Figures and Tables

**Table 1:** Summary of tissue specific trisomy 13 mosaicism.

Tissue type	# of nuclei with disomy 13	# of nuclei with trisomy 13	# of total nuclei	% of nuclei with trisomy 13
Umbilical cord	21	29	50	58%
Amnion	18	24	42	57%
Intermediate trophoblast	26	46	72	64%
Villous trophoblast	28	13	41	32%
Villous stroma	23	20	43	46%
